# Insulin Resistance Predicts Atherogenic Lipoprotein Profile in Nondiabetic Subjects

**DOI:** 10.1155/2017/1018796

**Published:** 2017-08-22

**Authors:** Flávia De C. Cartolano, Gabriela D. Dias, Maria C. P. de Freitas, Antônio M. Figueiredo Neto, Nágila R. T. Damasceno

**Affiliations:** ^1^Department of Nutrition, School of Public Health, University of Sao Paulo, São Paulo, SP, Brazil; ^2^Department of Experimental Physics, Institute of Physics, University of Sao Paulo, São Paulo, SP, Brazil

## Abstract

**Background:**

Atherogenic diabetes is associated with an increased cardiovascular risk and mortality in diabetic individuals; however, the impact of insulin resistance (IR) in lipid metabolism in preclinical stages is generally underreported. For that, we evaluated the capacity of IR to predict an atherogenic lipid subfraction profile.

**Methods:**

Complete clinical evaluation and biochemical analysis (lipid, glucose profile, LDL, and HDL subfractions and LDL phenotype and size) were performed in 181 patients. The impact of IR as a predictor of atherogenic lipoproteins was tested by logistic regression analysis in raw and adjusted models.

**Results:**

HDL-C and Apo AI were significantly lower in individuals with IR. Individuals with IR had a higher percentage of small HDL particles, lower percentage in the larger ones, and reduced frequency of phenotype A (IR = 62%; non-IR = 83%). IR individuals had reduced probability to have large HDL (OR = 0.213; CI = 0.999–0.457) and had twice more chances to show increased small HDL (OR = 2.486; CI = 1.341–7.051). IR was a significant predictor of small LDL (OR = 3.075; CI = 1.341–7.051) and atherogenic phenotype (OR = 3.176; CI = 1.469–6.867).

**Conclusion:**

IR, previously DM2 diagnosis, is a strong predictor of quantitative and qualitative features of lipoproteins directly associated with an increased atherogenic risk.

## 1. Introduction

The negative impact of diabetes in cardiovascular risk factors, atherogenesis, and cardiovascular events is well stablished in literature; however, the role of insulin resistance (IR) previously DM2 diagnosis is not totally clear. Clinically, it was defined as the inability of glucose uptake and utilization by insulin-dependent tissues and reduced insulin sensitivity, being the basis of type 2 diabetes mellitus (DM2) [1–3]. Almost 415 million people around the world are suffering from DM2, and recent data estimated that 318 million adults have impaired glucose tolerance and IR is prevalent in 20 to 30% of general population and in 90% of patients with DM2 [4]. In 2014, 11.9 million of Brazilian lived with DM2 and, by 2035, it is estimated that this prevalence will increase to 19.2 million [5].

IR is linked with hypertriglyceridemia and high-density lipoprotein cholesterol (HDL-C) [6–8]; however, the relationship with low-density lipoprotein cholesterol (LDL-C) is contradictory [3]. These findings can be explained by compensatory hyperinsulinemia due to IR, which induces increased free fatty acid (FFA) efflux from adipose tissue, thus raising VLDL production in the liver and, consequently, plasma triacylglycerol (TAG) and also reducing HDL-C by activation of cholesterol ester transfer protein (CETP) and increased clearance by the kidneys [9, 10].

Recently, Li et al. [10] described the complex bidirectional relationship of lipoprotein homeostasis and IR. HDL acts in both, IR and *β*-cell function, improving insulin secretion, increasing insulin sensitivity in the target tissues (adipose and muscle cells), and promoting positive effects on *β*-cell survival. This relation was confirmed by the association of qualitative and quantitative parameters of lipoprotein and DM2 [11, 12]. Up to now, few studies had analyzed lipoprotein subfractions in IR individuals before DM2 development [13, 14].

Regarding this background, the aim of this study was to compare the impact of IR effect on lipid metabolism and to evaluate if IR is a predictor of atherogenic lipoprotein profile in Brazilian individuals with IR and without DM2.

## 2. Methods

### 2.1. Subjects and Study Design

One hundred eighty-one adults of both genders were selected. Individuals were recruited from the University Hospital of the University of Sao Paulo. Subjects included in the study were 30 to 74 years old, without cardiovascular events (assessed by ECG and clinical evaluation) and without diagnosis of DM1 and DM2. Presence of DM was firstly evaluated by a direct interview using a structured questionnaire in which medical diagnosis of DM2 and current use of insulin and/or hypoglycemic drugs were self-reported by individuals. After, fasting glucose and insulin were analyzed to confirm DM2 diagnosis. If the fasting glucose level was close to the cut-off point (≥7.0 mmol/L), a second analysis was performed to confirm DM2, as recommended by Brazilian Diabetes Society [15]. Pregnant or lactating women, individuals who participate in other studies, illicit drug users, and alcoholics were not enrolled in this protocol. This study was approved by the Ethic Committee in Research of the University Hospital (n° 1126/11) and the School of Public Health, University of Sao Paulo (n° 2264). All subjects gave their written informed consent to participate and have their data published.

### 2.2. Demographic, Clinical, and Anthropometric Features

Demographic and clinical profile was evaluated using a structured questionnaire addressing gender, age, clinical information, family history of chronic diseases (father and mother), smoking status, blood pressure, and regular medication use.

From weight and height measures, body mass index (BMI) was calculated as weight (kg) divided by the square of the standing height (m^2^). The waist circumference (WC) and body composition (BIA) (Analyzer® model Quantum II, RJL Systems, Michigan, USA) were also evaluated.

### 2.3. Biochemical Analysis

After 12 h of fasting, blood samples were collected in vacutainer tubes containing ethylenediaminetetraacetic acid (EDTA) (1.0 *μ*g/mL). The protease inhibitors aprotinin (10.0 *μ*g/mL), benzamidine (10.0 *μ*M), and phenylmethylsulfonyl fluoride (PMSF) (5.0 *μ*M) plus the antioxidant butylated hydroxytoluene (BHT) (100.0 *μ*M) were added to the samples. Plasma and serum were separated by centrifugation at 3000 rpm for 10 minutes at 4°C, and samples were kept frozen at −80°C until analysis.

Plasma triacylglycerol (TAG), total cholesterol (TC), and HDL-C levels were measured using commercial kits from Labtest® (Minas Gerais, Brazil). Low-density lipoprotein cholesterol (LDL-C) level was calculated using the Friedewald equation [16]. Non-HDL was calculated from TC minus HDL-C. The apolipoprotein B (APO B) and apolipoprotein AI (APO AI) were determined by standard methods, using the autokit APO A1 and APO B® (Wako Chemicals USA Inc., Richmond, VA, USA). Glucose level was analyzed by an enzymatic and colorimetric kit (Glucose PAP Liquiform®, Labtest, Minas Gerais, Brazil). The insulin was performed by a commercial kit Insulin Human Direct ELISA Kit® (Life Technologies, Grand Island, NY). Insulin resistance (IR) was calculated with the homeostasis model assessment-insulin resistance (HOMA-IR) as follows: [fasting insulin concentration (*μ*U/mL) × fasting glucose (mmol/L)/22.5] [17]. IR classification was performed according to Stern et al. [18] that takes account HOMA-IR and BMI values: HOMA-IR > 4.65 or BMI > 28.90 kg/m^2^ or HOMA > 3.60 and BMI > 27.50 kg/m^2^. Based in these criteria, individuals were divided into the IR group and non-IR group.

The lipoprotein fractions (VLDL and IDL) and subfractions (HDL and LDL) were determined by the Lipoprint^®^ system. The LDL1 and LDL2 subfractions were classified as LDL_LARGE_, and subfractions LDL3 to LDL7 as smaller and denser particles (LDL_SMALL_). For HDL, ten subfractions were identified: HDL_LARGE_ (HDL1 to HDL3), HDL_INTERMEDIATE_ (HDL4 to HDL7), and HDL_SMALL_ (HDL8 to HDL10). The LDL phenotypes were based in cut-off points (phenotype A ≥ 268 Å and phenotype non-A < 268 Å). The mean LDL size was determined. All analyses were conducted in duplicate, and coefficients of variance intra- and interassay were 1–15%.

### 2.4. Statistical Analysis

Statistical analysis was performed using the Statistical Package for the Social Sciences (SPSS^®^), version 20.0. A two-sided *p* value <0.05 was considered statistically significant. The Kolmogorov-Smirnov test (*p* > 0.05) was applied to assess normality of data. Continuous variables with normally distributed data are presented as mean values and standard deviations (SD) while nonnormally distributed data are presented as medians and 25th and 75th percentiles. Categorical variables are presented as absolute values (*n*) and percentage (%). The comparison between groups was performed using Student's *t*-test for normally distributed data. Nonnormally distributed data were analyzed using the nonparametric Mann–Whitney *U* test. Categorical variables were compared using Pearson's chi-square test. To identify the effect of IR on lipoprotein subfraction profile, univariate logistic regression analysis was performed using the IR as an independent factor. Afterward, variables that showed correlations with IR values (*p* < 0.20) have been included into a multivariate logistic regression analysis. HDL1, HDL4, and HDL10 did not match this criterion. Model A which included age and gender as covariate and additional adjustment, respectively, was made for smoking, statin, and/or fibrate use in model B. Adjusted odds ratio (AOR) and 95% confidence interval (CI) were determined.

## 3. Results

The average age was 51.3 (12.0) years old for the non-IR group and 52.5 (9.3) years old for the IR group. About 60% of the subjects were women, and both groups showed similar frequency of smoking (*p* = 0.113). As expected, the IR group presented higher values for weight, BMI, and WC (Table 1).

HDL-C, APO AI, and LDL size levels were significantly lower in the IR group, which showed a higher TAG/HDL ratio. Over 80% of non-IR subjects exhibited phenotype A versus 62% in the IR group (*p* = 0.003) (Table 2).

The distribution of lipoprotein subfractions (Table 3) shows that the IR group had a higher percentage of intermediate (HDL5, HDL6, and HDL7) and small HDL particles (HDL 8) and lower percentage of large particles (HDL2 and HDL3), contributing to decrease in HDL_LARGE_ in the IR group. VLDL and LDL2 subfractions in the IR group showed higher percentages than those observed in non-IR subjects.

Presence of IR was associated with reduced chances to have HDL_LARGE_ (HDL2 and HDL3) and increased small particles (HDL8) and HDL_SMALL_. Regarding LDL subfractions, IR was a predictor of significant chance of increased LDL_SMALL_ (OR = 2.826; CI = 1.263–6.324) and phenotype non-A (OR = 3.011; CI = 1.424–6.366) (Table 4).

## 4. Discussion

This study showed that IR, without established DM2, is already a predictor of more atherogenic lipoprotein profile, contributing for a worse cardiovascular risk of Brazilian individuals.

IR is a key factor for the development of atherosclerosis and DM2, and the most common associated metabolic abnormality is high TAG and low HDL-C levels, whereas TC and LDL-C are not consistently altered [19].

Lipoproteins are heterogeneous structures, which vary in their size, density, and chemical composition and confer additional value to cholesterol content [20–22]. There are few studies that sought to analyze IR effects on lipid metabolism and lipoprotein subfractions in individuals without DM already clinically diagnosed [13, 14]. Recently, Shah et al. [23] described lipoprotein subclasses as a better marker to detect lipoprotein abnormalities in normoglycemic and prediabetic subjects.

Hypertriglyceridemia is considered the principal lipid abnormality in IR and plays a pivotal role in diabetic dyslipidemia [24–27]. This causal and strict relationship between IR and dyslipidemia was recently revised by Li et al. [10]. Our results confirmed that IR has negative effects on lipid metabolism and reinforces the connection between DM and CVD. Lower values of HDL-C, APO AI, higher TAG levels, TAG/HDL ratio, and percentage of VLDL observed in the IR group characterize a typical basis for atherogenic DM. This scenario evidences that changes in physical structure of lipoproteins start earlier to DM2 and reaffirms the negative impact of IR, highlighting the relevance of strategies focused in its prevention.

Elevated TAG levels are results of increased production and decreased clearance of TAG-rich lipoproteins in both fasting and nonfasting states [28]. These events favor increased production of VLDL, a prominent IR feature [29]. In agreement, our data showed that VLDL percentage in the non-IR group was lower than that in the IR group, confirming that individuals with IR were four times more likely to have lower HDL_LARGE_ percentage. This result was mainly due to differences in HDL2 and HDL3. Garvey et al. [12] using nuclear magnetic resonance (NMR) observed that progressive IR was associated with a decrease in HDL size, depletion of large HDL particles, and a modest rise in small HDL. They also described the increase in VLDL size and in large VLDL levels before and after multiple adjustment (age, BMI, and gender). Similarly, MacLean et al. [13] described in a small sample of obese insulin-resistance women that concentration of large HDL was lower, while HDL size was negatively correlated with plasma insulin. Our results expand this previous study, because it was based on a bigger sample and included both genders. In addition, our data show the concordance of results obtained by Lipoprint system and NMR [30].

The relationship between HDL size and cardiovascular risk is still a controversial issue. Clinical and epidemiological studies have shown that low HDL-C content is strongly and independently associated with cardiovascular diseases (CVD) [31]. However, some studies have questioned this association, hypothesizing that it requires a more specific analysis of this lipoprotein [32]. There is an important heterogeneity among HDL subfractions, comprising from small to large HDL, and different HDL subfractions appear to be associated with different functions. The HDL analysis performed on the JUPITER study suggested that concentration of HDL particles, rather than cholesterol content in the lipoprotein, was a more robust predictor of CVD events and a more appropriate target for therapeutic interventions [33].

According to Pirillo et al. [34], large HDL particles are more competent in reverse cholesterol transport (RCT), classically described as the primary physiological function of HDL [35], which represents the capacity to transfer excess cholesterol from peripheral cells to the liver for excretion, contributing to attenuate the atherosclerotic stimulus. However, recent studies have shown that small HDL particles have greater antioxidant and anti-inflammatory properties, preventing LDL oxidation in the subendothelial layer through reactive oxygen species (ROS) action [36].

Although there were controversial results in the literature, the majority of studies have associated lower concentrations of large HDL with cardiovascular events, dyslipidemia, obesity, metabolic syndrome, and diabetes [37].

Plausible biological mechanisms supporting the role of IR in HDL have been recently reviewed [26, 27]. The decrease in HDL-C induced by IR is associated with increased catabolism of this lipoprotein. This event involves the elevated transfer of TAG to HDL owing to hypertriglyceridemia caused by increased VLDL hepatic synthesis and decreased lipoprotein lipase (LPL) activity. This process is modulated by CETP, which leads formation of HDL rich in TAG, turning this lipoprotein a good substrate for the hepatic lipase enzyme (HL), responsible for HDL catabolism [26]. Our results corroborate with the negative influence of IR in the connection between TAG and HDL-C levels. In addition, the odds values also confirmed that IR was a predictor for higher VLDL and small HDL and lower large HDL particles. Possibly, these changes in HDL subfractions modify its functionality, reducing its antiatherogenic role.

In addition of the significant effect of IR on classical lipid profile, VLDL, and HDL subfractions, our data also showed that IR was related with increased chances to have smaller LDL. However, these structural changes were not related with modifications in total APO B and LDL-C levels, confirming that distribution of LDL and particle size adds information to classical evaluation of cholesterol and APO B content in this lipoprotein [38]. Small LDL particles are cited in several studies for their link with atherosclerosis [39].

Our results disclose that before DM2 diagnosis, the presence of IR can already cause multiple changes in lipids and structure of lipoproteins and it is supported by the worse LDL phenotype in the IR-group. Classically, two LDL phenotypes, named A and B, based on LDL dense and size are described [20, 21]. In our study, phenotype non-A (more atherogenic profile) was present in 17% of non-IR subjects, whereas this percentage was 38% in the IR group. Previous studies did not describe the negative impact of IR in these phenotypes. Thus, the higher prevalence of phenotype non-A observed in our study contributes to add more information regarding a more atherogenic lipoprotein profile when associated with higher TAG levels and small HDL particles. These results are particularly relevant because phenotypes obtained by Lipoprint system showed high concordance with other validated methods such as NMR (phenotype A = 100% and phenotype B = 75%) and Zaxis (phenotype A = 100% and phenotype B = 95%) [40].

Assessment of IR using the HOMA-IR equation is a simple and nonexpensive tool able to be used in clinical trials and in clinical practice routine. It has a high concordance level with hyperinsulinemic-euglycemic clamp technique, accepted as the gold standard method for IR diagnosis [41, 42]. However, some studies had demonstrated that HOMA-IR cut-off points are gender and age specific [43], while Gayoso-Diz et al. showed that metabolic syndrome components should be considered for IR classification [44]. These aspects suggest that HOMA-IR data should be analyzed with caution.

Based in these interactions, our odds ratios were adjusted by age and sex; however, few influences were detected by these confounders. The cut-off point used in our study was based on a previous large-population study that included Caucasian (European subjects from 17 cities), Mexican American, and Pima Indian [18]. Stern et al. [18] demonstrated that HOMA-IR isolated and/or combined with BMI could identify individuals with IR, previously DM2 diagnosis. This model is adequate for clinical trials due its high capacity to identify properly individuals with IR (specificity of 92% for HOMA-IR>4.65) but can also be used in clinical practice routine when BMI could be included (HOMA-IR > 3.6 and BMI > 27.5 kg/m^2^) or analyzed alone (BMI > 28.9 kg/m^2^). Additional advantage of this model is the ability to identify IR based only on one measure of BMI. This aspect is particularly relevant for individuals and population in developing countries where health systems are unable to diagnose early dysglycemia, and the incidence of DM shows accelerated growth. Though previous studies have proposed lesser cut-off points (<2.0), values adopted in our study were due to similar ethnic between individuals (Caucasian and Mexican American), and opposite to other studies, cut-off points proposed by Stern et al. [18] were validated by euglycemic insulin clamp technique in a large population-based study. Indeed, many studies described cut-off points ranging from 1.55 to 3.8 when different populations are analyzed, with different health status and distinct statistical approaches (ROC and percentiles), frequently based on a cross-sectional design. Recently, Lee et al. based on a large Chinese transversal and prospective study with 15 years of follow-up proposed cut-off points for dysglycemia (1.4) and DM2 (2.0) [45]. Despite the relevant results described, these cut-off points were not validated by euglycemia clamp technique and were based only on one glucose analysis. In addition to these aspects, the ethnicity exerts influence in IR as previously described [46, 47], explaining, in part, the different cut-off points described in literature [44]. In our study, most individuals were Caucasian and only 1.7% (*n* = 3) were Japanese Brazilian. Despite that, we decided not to exclude these individuals because they did not change our results' profile and this ethnic distribution is a representative of Brazilian population.

Altogether, clinical evaluation of IR can predict future changes in lipid metabolism and its impact in the development of CVD. These results are particularly relevant because they highlight the negative impact of IR, previously DM2 diagnosis, in qualitative aspects of lipid metabolism and cardiovascular risk not described previously in literature.

Finally, despite of the robust predictive role of IR on atherogenic lipoprotein profile observed in our study, we assume potential limitations of these results. First, criteria used to define DM could include additional analysis such as glucose tolerance test; however, in order to avoid this costly and time-consuming technique, we performed a rigorous and direct interview addressing specific questions about DM. The diagnosis was confirmed by fasting glucose, repeating it in cases where diagnosis was not clear. Second, some individuals included in our study were under statin/fibrate treatment. In this case, all groups were matched and the individuals enrolled should be under the same drug protocol at least 30 days prior to data collection. Third, the criteria used to determine IR were not a gold standard accepted as the hyperinsulinemic-euglycemic clamp, tolerance test glucose minimal model of Bergman, hyperglycemic clamp, or oral tolerance glucose/meal test. However, our goal was to identify the capacity of IR to predict atherogenic lipoprotein using a fast, simple, and low-cost tool applicable to clinical practice such as HOMA-IR.

In conclusion, our results showed that IR is associated with significant changes in quantitative and qualitative aspects of lipoproteins and it is a robust predictor of atherogenic lipoprotein profile in nondiabetic subjects.

## Figures and Tables

**Table 1:** Demographic, clinical profile, and anthropometry of the subjects according to the presence of insulin resistance.

Variables	Total (*n* = 181)	Non-IR group (*n* = 64)	IR group (*n* = 117)	*p*
Women (*n*, %)	110.0 (60.8)	40.0 (62.5)	70.0 (59.8)	0.725
Age, years (mean, SD)	52.1 (10.3)	51.3 (12.0)	52.5 (9.3)	0.476
Weight, kg (mean, SD)	80.1 (16.9)	67.4 (10.6)	87.0 (15.7)	**<0.001**
BMI, % (mean, SD)	30.1 (5.5)	25.0 (2.6)	32.9 (4.6)	**<0.001**
WC, cm (mean, SD)	98.3 (13.0)	86.1 (7.4)	105.0 (10.2)	**<0.001**
FM, % (mean, SD)	35.2 (11.9)	29.5 (9.8)	38.3 (11.9)	**<0.001**
SBP, mmHg (mean, SD)	134.3 (19.5)	130.2 (21.5)	136.6 (18.0)	**0.004**
DBP, mmHg (mean, SD)	82.0 (10.2)	78.5 (10.2)	83.9 (9.7)	**<0.001**
Smoking (*n*, %)	34.0 (18.8)	16.0 (25.0)	18.0 (15.4)	0.113
Statin (*n*, %)	45.0 (24.9)	15.0 (23.4)	30.0 (25.6)	0.743
Fibrate (*n*, %)	4.0 (2.2)	1.0 (1.6)	3.0 (2.6)	0.661

Data presented as mean (standard deviation) or absolute value (frequency). Comparative analysis for categorical variables was performed by Pearson's chi-square test (*p* < 0.05), and continuous variables were performed by Student's *t*-test (*p* < 0.05). BMI: body mass index; WC: waist circumference; SBP: systolic blood pressure; DBP: diastolic blood pressure; non-IR group: individuals without insulin resistance; IR group: individuals with insulin resistance.

**Table 2:** Biochemical profile of the subject according presence of insulin resistance.

Variables	Total (*n* = 181)	Non-IR group (*n* = 64)	IR group (*n* = 117)	*p*
TC (mmol/L)	5.38 (1.01)	5.43 (0.91)	5.35 (1.06)	0.591
HDL-C (mmol/L)	0.98 (0.28)	1.09 (0.26)	0.93 (0.28)	**0.001**
LDL-C (mmol/L)	3.67 (0.91)	3.72 (0.80)	3.62 (1.04)	0.495
TAG (mmol/L)	1.37 (1.03; 1.99)	1.15 (0.97; 1.50)	1.52 (1.14; 2.16)	**<0.001**
Glucose (mmol/L)	5.33 (8; 5.7)	5.1 (4.8; 5.3)	5.4 (5.1; 5.8)	**<0.001**
Insulin (*μ*U/mL)	17.0 (7.0)	12.0 (3.0)	20.0 (7.0)	**<0.001**
HOMA-IR	4.2 (1.9)	2.8 (0.7)	5.0 (2.0)	**<0.001**
TAG/HDL-C	1.5 (1.0; 2.3)	1.9 (0.8; 1.5)	1.6 (1.2; 2.6)	**<0.001**
Non-HDL-C (mmol/L)	4.42 (0.95)	4.38 (0.91)	4.42 (0.98)	0.686
APO AI (g/L)	1.33 (0.27)	1.42 (0.24)	1.29 (0.28)	**0.001**
APO B (g/L)	1.05 (0.23)	1.04 (0.23)	1.05 (0.23)	0.752
LDL size (Å)	270.0 (267.0; 272.0)	271.0 (269.0; 272.0)	270.0 (265.0; 272.0)	**0.045**
Phenotype A (*n*, %)	125.0 (69.0)	53.0 (83.0)	72.0 (62.0)	**0.003**

Data presented as mean (standard deviation) and median (p25-p75). Comparative analysis was performed by Student's *t*-tests or Mann–Whitney *U* test (*p* < 0.05). TC: total cholesterol; HDL-C: high-density lipoprotein cholesterol; LDL-C: low-density lipoprotein cholesterol; TAG: triacylglycerol; TAG/HDL-C: ratio between TAG and HDL-C; APO AI: apolipoprotein AI; APO B: apolipoprotein B; non-IR group: individuals without insulin resistance; IR group: individuals with insulin resistance.

**Table 3:** Distribution of lipoprotein subfractions of the subjects according to the presence of insulin resistance.

Variables (%)	Total (*n* = 181)	Non-IR group (*n* = 64)	IR group (*n* = 117)	*p*
VLDL	17.85 (3.93)	16.80 (3.29)	18.42 (4.15)	**0.017**
IDL	28.64 (4.09)	29.08 (3.54)	28.39 (4.36)	0.277
LDL1	16.88 (3.99)	17.56 (3.61)	16.51 (4.16)	0.089
LDL2	9.84 (4.01)	8.84 (3.65)	10.39 (4.10)	**0.013**
LDL3	2.48 (2.75)	1.78 (2.00)	2.87 (3.02)	0.062
LDL4	0.42 (1.09)	0.28 (1.09)	0.50 (0.36)	0.063
LDL5	0.06 (0.40)	0.07 (0.46)	0.05 (0.36)	0.931
LDL6	0.01 (0.14)	0.03 (0.24)	0.00 (0.00)	0.176
LDL7	0.00 (0.00)	0.00 (0.00)	0.00 (0.00)	1.000
LDL_LARGE_	2.97 (3.83)	2.16 (3.20)	3.42 (4.07)	0.081
LDL_SMALL_	26.72 (4.81)	26.40 (4.92)	26.89 (4.77)	0.515
HDL1	10.66 (3.61)	11.00 (3.57)	10.47 (3.64)	0.346
HDL2	12.32 (4.36)	14.33 (4.15)	11.23 (4.08)	**<0.001**
HDL3	7.34 (2.11)	8.32 (2.08)	6.81 (1.94)	**<0.001**
HDL4	9.37 (1.60)	9.45 (1.39)	9.33 (1.70)	0.619
HDL5	11.06 (1.61)	10.58 (1.55)	11.32 (1.59)	**0.003**
HDL6	21.66 (3.01)	20.31 (2.76)	22.40 (2.88)	**<0.001**
HDL7	7.72 (1.43)	7.37 (1.33)	7.92 (1.45)	**0.013**
HDL8	7.64 (1.73)	7.23 (1.58)	7.87 (1.78)	**0.017**
HDL9	5.98 (1.77)	5.64 (1.61)	6.16 (1.83)	0.056
HDL10	6.22 (3.98)	5.75 (3.62)	6.48 (4.15)	0.239
HDL_LARGE_	30.32 (8.45)	33.64 (8.66)	28.51 (7.80)	**<0.001**
HDL_INTERMEDIATE_	49.81 (4.87)	47.72 (4.72)	50.95 (4.58)	**<0.001**
HDL_SMALL_	19.84 (6.50)	18.63 (6.10)	20.51 (6.64)	0.063

Data presented as mean (standard deviation). Comparative analysis was performed by Student's *t*-tests or Mann–Whitney *U* test (*p* < 0.05). VLDL: very low-density lipoprotein; IDL: intermediate density lipoprotein; HDL: high-density lipoprotein; LDL: low-density lipoprotein; non-IR group: individuals without insulin resistance; IR group: individuals with insulin resistance.

**Table 4:** Multivariate logistic regression for effect of insulin resistance on lipoprotein subfraction profile.

Variables (%)	Raw data		Model A		Model B	
	OR	95% CI	AOR	95% CI	AOR	95% CI
*VLDL*						
Non-IR group	1.000		1.000		1.000	
IR group	1.974	[0.921; 4.230]	2.205	[0.965; 5.038]	2.332	[1.007; 5.404]

*HDL2*						
Non-IR group	1.000		1.000		1.000	
IR group	0.283	[0.141; 0.571]	0.267	[0.129; 0.552]	0.230	[0.108; 0.493]

*HDL3*						
Non-IR group	1.000		1.000		1.000	
IR group	0.322	[0.160; 0.645]	0.302	[0.146; 0.625]	0.298	[0.142; 0.625]

*HDL5*						
Non-IR group	1.000		1.000		1.000	
IR group	1.850	[0.881; 3.884]	1.843	[0.873; 3.890]	1.897	[0.890; 4.044]

*HDL6*						
Non-IR group	1.000		1.000		1.000	
IR group	3.367	[1.460; 7.766]	3.726	[1.579; 8.793]	3.936	[1.631; 9.498]

*HDL7*						
Non-IR group	1.000		1.000		1.000	
IR group	1.926	[0.919; 4.038]	1.927	[0.917; 4.047]	1.980	[0.930; 4.216]

*HDL8*						
Non-IR group	1.000		1.000		1.000	
IR group	2.305	[1.504; 5.039]	2.305	[1.042; 5.098]	2.363	[1.025; 5.445]

*HDL9*						
Non-IR group	1.000		1.000		1.000	
IR group	1.631	[0.772; 3.446]	1.367	[0.771; 3.474]	1.755	[0.806; 3.822]

*HDL*_*LARGE*_						
Non-IR group	1.000		1.000		1.000	
IR group	0.249	[0.123; 0.505]	0.232	[0.111; 0.484]	0.213	[0.099; 0.457]

*HDL*_*INTERMETDIATE*_						
Non-IR group	1.000		1.000		1.000	
IR group	3.367	[1.460; 7.766]	3.843	[1.606; 9.197]	4.698	[1.855; 11.901]

*HDL*_*SMALL*_						
Non-IR group	1.000		1.000		1.000	
IR group	2.400	[1.099; 5.239]	2.429	[1.110; 5.319]	2.486	[1.115; 5.543]

*LDL*_*SMALL*_						
Non-IR group	1.000		1.000		1.000	
IR group	2.826	[1.263; 6.324]	2. 794	[1.237; 6.310]	3.075	[1.341; 7.051]

*Phenotype non-A*						
Non-IR group	1.000		1.000		1.000	
IR group	3.011	[1.424; 6.366]	3.001	[1.404; 6.416]	3.176	[1.469; 6.867]

*n* = 181. Model A: adjusted for gender and age. Model B: adjusted for gender, age, smoking, statin, and fibrate. VLDL: very low-density lipoprotein; HDL-C: high-density lipoprotein cholesterol; non-IR group: individuals without insulin resistance; IR group: with insulin resistance.

## Abbreviations

Apo B: Apolipoprotein B
BHT: Butylated hydroxytoluene
BIA: Bioimpedance electric
BMI: Body mass index
CETP: Cholesterol ester transfer protein
CVD: Cardiovascular disease
DM2: Type 2 diabetes mellitus
ECG: Electrocardiogram
EDTA: Ethylenediaminetetraacetic acid
FFA: Free fatty acid
HDL: High-density lipoprotein
HDL-C: High-density lipoprotein cholesterol
HOMA-IR: Homeostasis model assessment-insulin resistance
IDL: Intermediate density lipoprotein
IR: Insulin resistance
LDL: Low-density lipoprotein
LDL-C: Low-density lipoprotein cholesterol
LPL: Lipoprotein lipase
NMR: Nuclear magnetic resonance
PMSF: Phenylmethylsulfonyl fluoride
RCT: Reverse cholesterol transport
TAG: Triacylglycerol
WC: Waist circumference.

## References

[1] Himsworth H. (1936). Diabetes mellitus: its differentiation into insulin sensitive and insulin insensitive types. *The Lancet*.

[2] Kahn C. R. (1994). Insulin action, diabetogenes, and the cause of type II diabetes. *Diabetes*.

[3] Reaven G. M. (2011). Insulin resistance: the link between obesity and cardiovascular disease. *The Medical Clinics of North America*.

[4] Ferrannini E. (1992). The insulin resistance syndrome. *Current Opinion in Nephrology & Hypertension*.

[5] International Diabetes Federation (2014). *IDF Diabetes Atlas*.

[6] Ferrannini E., Haffner S. M., Mitchell B. D., Stern M. P. (1991). Hyperinsulinaemia: the key feature of a cardiovascular and metabolic syndrome. *Diabetologia*.

[7] Reaven G. M. (1995). Pathophysiology of insulin resistance in human disease. *Physiological Reviews*.

[8] Unger R. H. (2008). Reinventing type 2 diabetes: pathogenesis, treatment, and prevention. *The Journal of the American Medical Association*.

[9] Reaven G. (2012). Insulin resistance and coronary heart disease in nondiabetic individuals. *Arteriosclerosis, Thrombosis, and Vascular Biology*.

[10] Li N., Fu J., Koonen D. P., Kuivenhoven J. A., Snieder H., Hofker M. H. (2014). Are hypertriglyceridemia and low HDL causal factors in the development of insulin resistance?. *Atherosclerosis*.

[11] Mackey R. H., Mora S., Bertoni A. G. (2015). Lipoprotein particles and incident type 2 diabetes in the multi-ethnic study of atherosclerosis. *Diabetes Care*.

[12] Garvey W. T., Kwon S., Zheng D. (2003). Effects of insulin resistance and type 2 diabetes on lipoprotein subclass particle size and concentration determined by nuclear magnetic resonance. *Diabetes*.

[13] MacLean P. S., Vadlamudi S., MacDonald K. G., Pories W. J., Houmard J. A., Barakat H. A. (2000). Impact of insulin resistance on lipoprotein subpopulation distribution in lean and morbidly obese nondiabetic women. *Metabolism*.

[14] Festa A., Williams K., Hanley A. J. (2005). Nuclear magnetic resonance lipoprotein abnormalities in prediabetic subjects in the Insulin Resistance Atherosclerosis Study. *Circulation*.

[15] Milech A., Angelucci A. P., Golbert A., Carrilho A. J., Ramalho A. C., Aguiar A. C. (2016). *Diretrizes da Sociedade Brasileira de Diabetes (2015-2016)*.

[16] Friedewald W. T., Levy R. I., Fredrickson D. S. (1972). Estimation of the concentration of low-density lipoprotein cholesterol in plasma, without use of the preparative ultracentrifuge. *Clinical Chemistry*.

[17] Matthews D. R., Hosker J. P., Rudenski A. S., Naylor B. A., Treacher D. F., Turner R. C. (1985). Homeostasis model assessment: insulin resistance and beta-cell function from fasting plasma glucose and insulin concentrations in man. *Diabetologia*.

[18] Stern S. E., Williams K., Ferrannini E., DeFronzo R. A., Bogardus C., Stern M. P. (2005). Identification of individuals with insulin resistance using routine clinical measurements. *Diabetes*.

[19] DeFronzo R. A., Ferrannini E. (1991). Insulin resistance: a multifaceted syndrome responsible for NIDDM, obesity, hypertension, dyslipidemia, and atherosclerotic cardiovascular disease. *Diabetes Care*.

[20] Packard C., Caslake M., Shepherd J. (2000). The role of small, dense low density lipoprotein (LDL): a new look. *International Journal of Cardiology*.

[21] Berneis K. K., Krauss R. M. (2002). Metabolic origins and clinical significance of LDL heterogeneity. *Journal of Lipid Research*.

[22] Diffenderfer M. R., Schaefer E. J. (2014). The composition and metabolism of large and small LDL. *Current Opinion in Lipidology*.

[23] Shah A. S., Davidson W. S., Gao Z., Dolan L. M., Kimball T. R., Urbina E. M. (2016). Superiority of lipoprotein particle number to detect associations with arterial thickness and stiffness in obese youth with and without prediabetes. *Journal of Clinical Lipidology*.

[24] Arca M., Pigna G., Favoccia C. (2012). Mechanisms of diabetic dyslipidemia: relevance for atherogenesis. *Current Vascular Pharmacology*.

[25] Steiner G., Vranic M. (1982). Hyperinsulinemia and hypertriglyceridemia, a vicious cycle with atherogenic potential. *International Journal of Obesity*.

[26] von Eckardstein A., Sibler R. A. (2011). Possible contributions of lipoproteins and cholesterol to the pathogenesis of diabetes mellitus type 2. *Current Opinion in Lipidology*.

[27] Drew B. G., Rye K. A., Duffy S. J., Barter P., Kingwell B. A. (2012). The emerging role of HDL in glucose metabolism. *Nature Reviews Endocrinology*.

[28] Wu L., Parhofer K. G. (2014). Diabetic dyslipidemia. *Metabolism*.

[29] Adiels M., Olofsson S. O., Taskinen M. R., Borén J. (2008). Overproduction of very low-density lipoproteins is the hallmark of the dyslipidemia in the metabolic syndrome. *Arteriosclerosis, Thrombosis, and Vascular Biology*.

[30] Otvos J. D., Jeyarajah E. J., Bennett D. W., Krauss R. M. (1992). Development of a proton nuclear magnetic resonance spectroscopic method for determining plasma lipoprotein concentrations and subspecies distributions from a single, rapid measurement. *Clinical Chemistry*.

[31] Barter P. J., Puranik R., Rye K. A. (2007). New insights into the role of HDL as an anti-inflammatory agent in the prevention of cardiovascular disease. *Current Cardiology Reports*.

[32] Sirtori C. R., Calabresi L., Franceschini G. (2001). Cardiovascular status of carriers of the apolipoprotein A-I(Milano) mutant: the Limone sul Garda study. *Circulation*.

[33] Rosenson R. S., Brewer H. B., Ansell B. (2013). Translation of high-density lipoprotein function into clinical practice: current prospects and future challenges. *Circulation*.

[34] Pirillo A., Norata G. D., Catapano A. L. (2013). High-density lipoprotein subfractions—what the clinicians need to know. *Cardiology*.

[35] Glomset J. A. (1968). The plasma lecithins:cholesterol acyltransferase reaction. *Journal of Lipid Research*.

[36] Kontush A., Chapman M. J. (2010). Antiatherogenic function of HDL particle subpopulations: focus on antioxidative activities. *Current Opinion in Lipidology*.

[37] Tian L., Jia L., Mingde F. (2006). Alterations of high density lipoprotein subclasses in obese subjects. *Lipids*.

[38] Sacks F. M., Campos H. (2003). Clinical review 163: cardiovascular endocrinology: low-density lipoprotein size and cardiovascular disease: a reappraisal. *The Journal of Clinical Endocrinology and Metabolism*.

[39] Lamarche B., Lemieux I., Després J. P. (1999). The small, dense LDL phenotype and the risk of coronary heart disease: epidemiology, pathophysiology and therapeutic aspects. *Diabetes & Metabolism*.

[40] Hoefner D. M., Hodel D. S., O’Brien J. F. (2001). Development of a rapid, quantitative method for LDL subfractionation with use of the quantimetrix lipoprint LDL system. *Clinical Chemistry*.

[41] Bonora E., Targher G., Alberiche M. (2000). Homeostasis model assessment closely mirrors the glucose clamp technique in the assessment of insulin sensitivity: studies in subjects with various degrees of glucose tolerance and insulin sensitivity. *Diabetes Care*.

[42] DeFronzo R. A., Tobin J. D., Andres R. (1979). The glucose clamp technique: a method for quantifying insulin secretion and resistance. *The American Journal of Physiology*.

[43] Gayoso-Diz P., Otero-González A., Rodriguez-Alvarez M. X. (2011). Insulin resistance index (HOMA-IR) levels in a general adult population: curves percentile by gender and age. The EPIRCE study. *Diabetes Research and Clinical Practice*.

[44] Gayoso-Diz P., Otero-González A., Rodriguez-Alvarez M. X. (2013). Insulin resistance (HOMA-IR) cut-off values and the metabolic syndrome in a general adult population: effect of gender and age: EPIRCE cross-sectional study. *BMC Endocrine Disorders*.

[45] Lee C. H., Shih A. Z. L., Woo Y. C. (2016). Optimal cut-offs of homeostasis model assessment of insulin resistance (HOMA-IR) to identify dysglycemia and type 2 diabetes mellitus: a 15-year prospective study in Chinese. *PLoS One*.

[46] McKeigue P. M., Shah B., Marmot M. G. (1991). Relation of central obesity and insulin resistance with high diabetes prevalence and cardiovascular risk in South Asians. *Lancet*.

[47] Banerji M. A., Faridi N., Atluri R., Chaiken R. L., Lebovitz H. E. (1999). Body composition, visceral fat, leptin, and insulin resistance in Asian Indian men. *The Journal of Clinical Endocrinology and Metabolism*.

